# Improving post-partum family planning services provided by female community health volunteers in Nepal: a mixed methods study

**DOI:** 10.1186/s12913-020-4969-1

**Published:** 2020-02-17

**Authors:** Kusum Thapa, Rolina Dhital, Sameena Rajbhandari, Sangeeta Mishra, Shanti Subedi, Bhogendra Raj Dotel, Sapana Vaidya, Saroja Pande, Emily-Anne Tunnacliffe, Anita Makins, Sabaratnam Arulkumaran

**Affiliations:** 1 Nepal Society of Obstetricians and Gynaecologists, Paropakar Maternity and Women’s Hospital, Thapathali, Kathmandu, GPO: 23700 Nepal; 2 Koshi Zonal Hospital, Biratnagar, Morang District, Province One Nepal; 3 Nobel Medical College Teaching Hospital, Biratnagar, Morang District, Province One Nepal; 4 Health Directorate-Province One, Dhankuta, Nepal; 5 International Federation of Obstetrics and Gynecology, London, UK

**Keywords:** Family planning services, Post-partum period, Contraception, Intrauterine devices, Community health workers

## Abstract

**Background:**

Family planning services in the post-partum period, termed post-partum family planning (PPFP) is critical to cover the unmet need for contraception, especially when institutional delivery rates have increased. However, the intention to choose PPFP methods such as post-partum intrauterine devices (PPIUD) remains low in countries such as Nepal. Community health workers such as Female Community Health Volunteers (FCHVs) could play an important role in improving the service coverage of PPFP in Nepal. However, their knowledge of PPFP and community-based services related to PPFP remain unclear. This study aims to assess the effect on community-based PPFP services by improving FCHV’s knowledge through orientation on PPFP.

**Methods:**

We conducted this mixed-methods study in Morang District in Nepal. The intervention involved orientation of FCHVs on PPFP methods. We collected quantitative data from three sources; via a survey of FCHVs that assessed their knowledge before and after the intervention, from their monthly reporting forms on counseling coverage of women at different stages of pregnancy from the communities, and by interviewing mothers in their immediate post-partum period in two selected hospitals. We also conducted six focus group discussions with the FCHVs to understand their perception of PPFP and the intervention. We performed descriptive and multivariable analyses for quantitative results and thematic analysis for qualitative data.

**Results:**

In total, 230 FCHVs participated in the intervention and their knowledge of PPFP improved significantly after it. The intervention was the only factor significantly associated with their improved knowledge (adjusted odds ratio = 24, *P* < 0.001) in the multivariable analysis. FCHVs were able to counsel 83.3% of 1872 mothers at different stages of pregnancy in the communities. In the two hospitals, the proportion of mothers in their immediate post-partum period whom reported they were counseled by FCHVs during their pregnancy increased. It improved from 7% before the intervention to 18.1% (*P* < 0.001) after the intervention. The qualitative findings suggested that the intervention improved their knowledge in providing PPFP counseling.

**Conclusion:**

The orientation improved the FCHV’s knowledge of PPFP and their community-based counseling. Follow-up studies are needed to assess the longer term effect of the FCHV’s role in improving community-based PPFP services.

## Background

Globally, there has been increasing recognition of the importance of providing contraceptive services to women immediately after childbirth. However, three in five women around the world face an unmet need for post-partum contraception in the first two years after childbirth [1]. The unmet need for post-partum family planning (PPFP) is much higher in low and middle-income countries (LMICs). In Nepal, one in two women face this unmet need during the post-partum period [2].

PPFP is defined as the prevention of unintended pregnancy and closely spaced pregnancies during the first 12 months following childbirth [3]. PPFP helps to space pregnancies and prevent unintended pregnancies but is often neglected by users and providers due to sociocultural influences and lack of knowledge and availability of services [4–6].

The copper intrauterine device (IUD) is a PPFP method that can be used in the immediate post-partum period. It can be used immediately after childbirth either within 10 min of placenta being removed or 48 h of childbirth and is termed post-partum IUD (PPIUD) [7]. Women who undergo cesarean section can also choose PPIUD where the route of insertion is intra-cesarean [8]. PPIUD can be used by women who want to limit or space their pregnancies, it can be used effectively for up to 12 years and if desired can be removed easily at any time after insertion with immediate return of fertility [9].

In Nepal, PPIUD is currently the only reversible PPFP method available in the immediate post-partum period. Despite the advantages of PPFP, the use of immediate PPFP methods such as PPIUD remains low in Nepal as compared to other countries [10]. A qualitative study from the earlier phase of implementation of this initiative identified societal norms, as well as peer and health provider influences as factors that affected women’s behavior on choosing a PPFP method [11]. The community level awareness of PPFP in general and in particular on PPIUD was very low [11].

In low-income countries, such as Nepal, many women continue to give birth at home and those who give birth in facilities often do not return for follow-up [12]. Nevertheless, the number of women giving birth in health facilities such as hospitals, health posts, and birthing centers has increased from 18% in 2006 to 57% in 2016 [13]. This increase in health facility deliveries provides a one stop approach for women to receive maternity care as well as immediate PPFP services [12].

### Institutionalization of PPFP/PPIUD initiative in Nepal

Since 2015, the government of Nepal and Nepal Society of Obstetricians and Gynecologists (NESOG) with the support from the International Federation of Gynecology and Obstetrics (FIGO) has implemented the institutionalization of immediate PPFP services initiative in six major referral hospitals across Nepal. The initial phases of the Initiative focused on PPIUD and the institutionalization of services of facility-based activities [14] including a time-limited employment of a counselor that improved PPFP counseling coverage. However, the overall uptake of immediate PPFP methods such as PPIUD remained lower than in other countries [12]. Moreover, a qualitative study conducted during an earlier phase of this initiative identified Female Community Health Volunteers (FCHVs) as an important influencer of women’s behavior in choosing a PPFP method [11]. However, FCHVs or community-based activities were not part of the implementation in the earlier phase of this initiative. Built upon the lessons learned from the earlier implementation, the initiative introduced community linkages through the involvement of FCHVs in 2018 within the catchment areas of two implementing hospitals in Morang District of Province One in Nepal. If successful, this intervention would provide a sustainable means of providing community-based PPFP counseling in the future.

### Role of FCHV in community linkage

The FCHV program was introduced in 1988 to support Nepal’s family planning (FP) program nationwide [15]. FCHVs are recognized as a pillar of the health system in Nepal and an important bridge linking the communities to health care services. They are often the first point of contact for people in their communities who are in need of health services. FCHVs are not certified health providers, instead, they are women of different educational levels selected within each community to volunteer and promote health in the communities. Currently, over 52,000 FCHVs are actively volunteering in Nepal with each FCHV reaching around 500 people in their communities [15]. All FCHVs undergo 18 days of basic training on FP, Maternal Newborn and Child Health (MNCH), and nutrition when they join this voluntary service [15].

For MNCH related health promotion activities, FCHVs conduct mothers’ group meetings in the communities every month and organize counseling sessions for pregnant women in the communities on FP, birth preparedness and the advantages of institutional delivery [16]. They also identify danger signs among pregnant and post-partum mothers and newborns through their antenatal and postnatal home visits, and refer any with complications to health facilities [16].

The involvement of FCHVs in community health interventions in Nepal has effectively improved population-based coverage for various health services [17–21]. A post disaster health promotion intervention study involving FCHVs in an earthquake affected district in Nepal showed that their involvement helped in improving the facility delivery among the mothers from 63 to 83% [21]. The same study also identified the improved odds of mothers having better knowledge and behaviors related to MNCH after the intervention [21]. Another cohort study found that those who received follow-up visits by FCHVs had 84% less relative risk of infant deaths due to low birth weight compared to those who had no FCHVs’ involvement [19]. Despite the FCHVs’ role in improving MNCH indicators in the country, their specific role in promoting PPFP was unclear. This study assessed the effect of an intervention in improving the knowledge of FCHVs on PPFP as a primary outcome. We also assessed the early effect of the intervention on PPFP counseling coverage in the communities and in the major referral hospitals as secondary outcomes.

## Methods

### Study design

This is a mixed methods study following the sequential explanatory method [22] where the qualitative study followed the quantitative study and helped in explaining the quantitative findings in more depth. The study utilized three quantitative and one qualitative techniques. The quantitative methods included knowledge assessment of FCHVs before and after the intervention, cross-sectional study of post-partum mothers admitted in the hospital, and the review of monthly reporting forms on FCHV’s service coverage. The qualitative method included focus group discussions (FGD) with the FCHVs. We followed consolidated criteria for reporting qualitative research (COREQ) to conduct FGD, analyze, and report the qualitative findings.

### Study settings

We conducted this study in two major referral hospitals and their catchment communities in Morang district, Province One. We assessed the knowledge of FCHVs in communities that were within the catchment areas of 23 peripheral health facilities in Morang District, Province One. In Nepal, FCHVs are linked with the peripheral health facilities in the communities, whereby each peripheral health facility usually supervises around nine to ten FCHVs in their communities and facilitate a monthly FCHV meeting in the health facilities to document their monthly activities [15, 16].

The selected 23 peripheral health facilities in this study are also the major catchment areas of the two major referral hospitals in Morang District namely, Koshi Zonal Hospital (KZH) and Nobel Medical College Teaching Hospital (NMCTH). Both hospitals have high obstetric workloads. Though KZH and NMCTH were the two major hospitals in the district, there are many other private hospitals, non-for profit hospitals, and birthing centers where women from the same district could go for childbirth. Similarly, the two study hospitals also provided obstetric services to a wider population of mothers from different regions across the country. However, these two hospitals were selected specifically for this study as these were the only two hospitals in Morang District that had implemented the FIGO and NESOG supported PPFP/PPIUD initiative. Some other birthing centers and a non-profit hospital in the region also provide PPFP/PPIUD services through the government, however, exact data is not available.

### Intervention

The intervention activities for this study were designed based on the findings from the previous qualitative study [11], and feedback from key stakeholders of MNCH and FP programs in Nepal. The intervention was focused on a cascade process of orientation of FCHVs in the selected communities. Under the leadership of Provincial Health Directorate in Province One, a pool of 15 facilitators were trained for the orientation of PPFP programs. The provincial level facilitators then trained 92 health providers at the peripheral health facility-level from 23 peripheral health facilities in Morang District, Province One. In December 2018, these health providers then trained nine to ten FCHVs within their peripheral health facility catchment communities, totaling 230 FCHVs.

The content and process of delivery in the training session remained the same for each level. The facilitators at each level employed a simplified approach using the FCHV User’s Guide developed by the government of Nepal. The facilitators delivered key messages on PPFP, displayed posters related to different methods of PPFP, and also demonstrated the examples and samples of PPFP methods. The contents covered different methods of PPFP along with the advantages and disadvantages of each method. The content focused on PPIUD in more detail as it was the only long acting reversible method available in the immediate post-partum period in Nepal.

The sessions were interactive in nature. The facilitators tested the knowledge of the FCHVs through a structured questionnaire on the five key messages for immediate PPFP to assess their knowledge before and after the intervention on the same day. The facilitators discussed the changes in FCHV’s scores and tried to address any areas of confusion.

Both FCHVs and health providers agreed to discuss the progress with PPFP related activities in their regular monthly meetings in the peripheral health facilities. The FCHVs were also required to maintain monthly reporting forms following the intervention and report on counseling coverage of PPFP for mothers at different stages of pregnancy within their communities. They were also required to report on the number of mothers who chose PPIUD after giving birth in various health facilities including the two study hospitals.

### Study participants

The study participants comprised of FCHVs and mothers in the immediate post-partum period from the two hospitals. FCHVs were from communities within the major catchment areas of the two referral hospitals. In order to be considered eligible for both the quantitative and qualitative studies, the FCHVs had to be residing within the same area during the time of intervention, and willing to participate in the intervention.

Mothers in their immediate post-partum period, who were still in hospital and willing to participate and were eligible for the quantitative study were selected through simple random sampling. The mothers who had suffered any major post-partum complication, those who were physically unable to answer the questions and those admitted to intensive care units were excluded from this study due to ethical reasons.

### Study tools

We used three different study tools for the quantitative study.

#### FCHVs’ pre-posttest questionnaire

For FCHVs, the quantitative study tool comprised of questions related to their general characteristics and their knowledge of PPFP (Additional file 1). The general characteristics included their age, level of education, number of years working as a FCHV, and their exposure to FP related trainings in the past. For knowledge, we adopted the questions from the FCHV User’s Guide on PPFP developed by the government of Nepal. The questionnaire had a list of five questions that assessed the key concepts of immediate PPFP and specifically PPIUD. The FCHVs had to choose either “true” or “false” for each question. Considering the possibilities of limited literacy of the FCHVs, the tools were designed with pictorial responses for each question. Others could read out the questions to FCHVs and they could choose a happy face for a “true” response and a sad face for a “false” response. The same tools were used before and after the intervention.

#### FCHV’s monthly reporting form on community counseling coverage

The FCHVs also provided their monthly reporting forms that included information on their PPFP counseling coverage in the post-intervention period between January and February 2019 (Additional file 2). The information included the total number of mothers at different stages of pregnancy in the communities and the proportion of these mothers counseled by FCHVs on PPFP. The numbers also represented the women who had already given birth after they were counseled and the proportion of the counseled mothers who chose PPIUD after giving birth in different facilities. The FCHVs obtained this information through individual interaction with the women in their communities.

#### Mothers’ interview questionnaire

We used mothers’ questionnaires to assess if the effect of community counseling coverage was reflected amongst the mothers giving birth in the two referral hospitals (Additional file 3). For the purpose of this study, we asked two specific questions to the mothers in their immediate post-partum period. The questions focused on whether they had ever interacted with FCHVs during their pregnancy and if they had ever been counseled by the FCHVs during their pregnancy. The mothers were required to answer either “yes” or “no” to these two questions.

For the qualitative study, the researchers conducted FGD with the use of FGD checklists (Additional file 4). The FGD checklists included questions related to the FCHVs’ knowledge of PPFP, their impression of the intervention, their community-based activities after the intervention and their recommendations.

### Study variables

The primary outcome was FCHVs’ knowledge of immediate PPFP. The intervention was the main exposure variable. The other covariates included FCHV’s age, level of education, number of years working as a FCHV, and their exposure to FP related orientations in the past.

The secondary outcome was the counseling coverage of PPFP by FCHVs. The counseling coverage in the communities was measured directly through FCHVs’ reports. It included the number of mothers at different stages of pregnancy being counseled by FCHVs and the uptake of PPIUD among the counseled mothers who had recently given birth in different facilities including the two hospitals. Not all the mothers counseled by FCHVs had given birth during the study period. Moreover, not all the mothers who had given birth had gone to the two referral hospitals as there are other health facilities in Morang District.

The counseling coverage was also measured indirectly with the aim to validate if counseling activities in the communities had started to be reflected amongst the mothers who gave birth in the two study hospitals. We measured if the mothers delivering in the hospitals had ever interacted with and been counseled by the FCHVs during their last pregnancy before they came for childbirth. The mothers delivering in the hospitals represented women from a wider population and not just limited to the FCHVs’ working areas.

### Sample size

For FCHVs, we calculated the size using the formula to compare two proportions [23]. We assumed that FCHVs would have a minimum of 22% knowledge before the intervention and would reach over 35% after the intervention. The minimum required sample size was calculated to be 201 for each phase of the intervention with 80% power for both groups to have significantly different proportions at 5% level of significance. However, considering incomplete response rates, the sample size was increased. In total data for 230 FCHVs was included in the analysis of both pre- and post-intervention data.

For post-partum mothers from the two referral hospitals, we used the same formula to compare the two proportions. We assumed that the intervention would increase the proportion of mothers being counseled by FCHVs by 5% to a minimum of 15% after the intervention. The minimum required sample size was calculated as 157 for each group before and after the intervention, with 80% power for both groups to have significantly different proportions at 5% level of significance. However, considering incomplete data and response rates, we increased the sample size to collect the data of 250 mothers from each hospital [23].

After initial review of the quantitative data, we purposively selected 6 groups of FCHVs to participate in the qualitative study until the information reached point of saturation.

### Data collection

We collected both quantitative and qualitative data between November 2018 and February 2019. The intervention for FCHVs was conducted in the month of December 2018 across 23 peripheral health facilities. The quantitative data to assess FCHVs’ knowledge of PPFP was collected before and after the orientation program on the same day of the intervention. Random sampling was not a feasible option, we therefore used convenience sampling to recruit FCHVs in the intervention as we had to coordinate with the peripheral health facilities to invite FCHVs.

We collected the data for FCHVs’ counseling coverage from two different sources. The FCHVs’ monthly reporting form for PPFP was introduced after the intervention and comprised of information from the post-intervention period only between January and February 2019.

The data from post-partum mothers in the facilities was collected before and after the intervention. The mothers recruited in the study during November and December 2018 were considered the pre-intervention group and those recruited during January and February 2019 were considered the post-intervention group. We used a simple random sampling method to recruit mothers in their immediate post-partum period. Every day the data collection officers (DCO) created a sampling frame of all the bed numbers with mothers in their immediate post-partum period who had given birth in the selected facilities. They then randomly selected two-bed numbers in each hospital through the opaque lottery method from the list of bed numbers and requested the mothers to participate in the study. If the selected mothers refused to participate, the DCOs repeated the process of random sampling leaving out the bed numbers of the mothers who had refused to participate. DCOs then interviewed the mothers who gave consent from the randomly selected bed numbers to participate in the study using a structured questionnaire on a mobile tablet.

The data collection for the qualitative study was conducted in late February 2019 and was guided by the quantitative study. After initial review of the quantitative results on FCHV’s knowledge and the counseling coverage, we purposively selected six peripheral health facilities where the intervention had taken place. Each facility represented a unique group of FCHVs who had participated in the intervention showing different levels of knowledge of PPFP. The FCHVs from the selected health facilities were approached via telephone and requested to be physically present in the health facilities to participate in the FGD on the assigned dates. All the FCHVs that were contacted attended a FGD. A well-trained female qualitative researcher (RD) moderated the FGD with assistance from a female note taker. All FGDs were conducted in closed meeting rooms of the respective health facilities. Only the moderator, note taker and the FCHVs were present in the FGDs to maintain confidentiality and reduce bias. The moderator first explained the objective of the FGD and introduced the research team to the participants. The FCHVs were familiar with the reasons for conducting the research and a relationship with the research team had already been established through their earlier participation in the intervention and quantitative study. The FGDs took around 40 to 45 min to complete. All FGDs were audio recorded and field notes were transcribed by hand. The moderator (RD) summarized the discussions based on the field notes with the FCHV for their feedback, to ensure that the research team had understood them correctly.

### Data analysis

We analyzed three different quantitative data sets using SPSS version 23 and the significance level was considered to be a *p* value of less than 0.05.

- For FCHVs, we conducted the descriptive analysis of their general characteristics, and bivariate and multivariable analysis to assess their knowledge of PPFP. We used chi-squared test to assess the change in proportions of FCHVs correctly answering questions on each of the five key messages on PPFP before and after the intervention. We used a logistic regression model to assess factors associated with their PPFP knowledge score. The outcome variable for knowledge was dichotomized to less than 4 correct answers and 4 or more correct answers out of 5 questions on key PPFP messages.
- For the counseling coverage by FCHVs in the communities, we conducted descriptive analysis.
- For the post-partum mothers at the two referral hospitals, we used a chi-squared test to assess the differences between the pre- and post-intervention groups on their responses to two questions; if they had ever interacted with a FCHV and if they had been counseled by an FCHV during pregnancy.
- We used thematic analysis [24] for qualitative data based on the five priori themes generated from the findings of quantitative data. All FGDs were conducted in Nepali and was translated into English language during the process of transcription. The transcripts were then reviewed by KT and SR and analyzed with Dedoose software version 8.0.42. KT, RD and SR first read and re-read the transcripts to familiarize themselves with the broad ideas and generated initial codes. KT, RD and SR then gathered all the coded data and collated the codes with the relevant priori themes. The themes were then reviewed by all the authors and assessed to determine whether the data was consistent with the findings and the overall objective of the study. KT, RD and SR then selected original and vivid quotes from FCHVs for each theme to provide more insights.

## Results

### Quantitative results

#### General characteristics of FCHVs orientated on PPFP

Table 1 shows the general characteristics of the FCHVs who attended the orientation on PPFP in Province One. Among the 230 FCHVs, the median age was 48 years [range 19–70 years]. The median years of working as an FCHV was 21 [range 2 months – 28 years]. In total, 45.7% of the FCHVs (*n* = 105) had primary level education, 33.9% (*n* = 78) had secondary level education, and 19.1% (*n* = 44) had no formal education though they were able to write their names and read some very basic information. The majority of the FCHVs (88.3%) had previously received a general orientation on different FP methods, however, the majority of them (73.9%) had never received orientation specifically related to PPFP.

**Table 1:** General characteristics of FCHVs oriented on PPFP

Characteristic of FCHVs	*N* = 230		
	Median	Minimum	Maximum
Age, in years	48	19	70
Years of working as FCHV, in years	21	< 1	28
	n (%)		
Education			
Cannot read and write	44 (19.1)		
Primary	105 (45.7)		
Secondary	78 (33.9)		
University	3 (1.3)		
FP orientation in the past			
No	27 (11.7)		
Yes	203 (88.3)		
PPFP orientation in the past			
No	170 (73.9)		
Yes	60 (26.1)		

#### Changes in knowledge scores on PPFP of FCHV before and after the orientation

Table 2 shows the proportion of FCHVs answering the PPFP questions correctly before and after the intervention. Prior to the orientation, the lowest score was observed for the first question on whether women can use a contraceptive method immediately after birth. The proportion of FCHVs answering this question correctly improved from 45.7 to 75.2% after the orientation. The second lowest score was observed for question 3 which stated that women who undergo a cesarean section can use PPIUD. The proportion of FCHVs answering this question correctly improved from 50.2 to 97.4% after the orientation. Overall, the proportion of FCHVs being able to answer 4 or more questions correctly improved from 46.5 to 95.2%.

**Table 2:** FCHVs Change in knowledge scores on PPFP

Knowledge of PPFP	Pre-orientation (*n* = 230)	Post-orientation (n = 230)	*P*-value
Key messages	n (%)	n (%)	
1. Immediately after delivery, mothers can use contraception	105 (45.7)	173 (75.2)	< 0.001
2. Post-partum Intrauterine devices can provide protection up to twelve years	205 (89.5)	255 (97.8)	< 0.001
3. Mothers who undergo a caesarean section can have post-partum IUD inserted	115 (50.2)	244 (97.4)	< 0.001
4. IUDs can be inserted immediately after giving birth	148 (64.3)	223 (97.0)	< 0.001
5. If IUD strings are seen outside vagina, they should go for follow-up immediately	180 (78.3)	215 (93.9)	< 0.001
Overall correct answers			
Less than 4 correct answers	123 (53.5)	11 (4.8)	< 0.001
4 or more correct answers	107 (46.5)	219 (95.2)	

#### Factors associated with improved knowledge scores among FCHV

Table 3 shows the logistic regression model assessing the factors associated with knowledge of PPFP among FCHVs. The knowledge of PPFP was divided into those with correct knowledge of less than 4 questions and those with correct knowledge of 4 or more questions. There was a 24 fold increase in FCHVs ability to correctly answer 4 or more questions (AOR = 24.0, *p* < 0.001). Other sociodemographic characteristics of FCHVs and their exposure to any previous FP related training had no significant association with their knowledge scores.

**Table 3:** Factors associated with knowledge scores^a^ on PPFP among FCHVs

	*N* = 460^b^		
Characteristics	AOR	95% CI	*P*-value
Intervention			
Pre-intervention^c^	1		
Post-intervention	24.0	(12.37–46.97)	< 0.001
Age	0.98	(0.96–1.02)	0.985
Years of working as FCHV	0.99	(0.96–1.03)	0.934
Education			
No formal education	1		
Primary level and higher	0.67	(0.40–1.11)	0.124
FP orientation in the past			
Yes	1.19	(0.73–5.01)	0.186
No^c^	1		
IUD orientation in the past			
Yes	1.04	(0.56–1.90)	0.897
No^c^	1		
PPFP orientation in the past			
Yes	0.951	(0.53–1.68)	0.862
No^c^	1		

^a^knowledge scores = less than 4 correct answers and 4 or more correct answers

^b^pooled data from pre- and post-intervention

^c^reference group

#### Community-based PPFP counseling by FCHVs

Table 4 shows data from the peripheral health facilities monthly meeting records, collected and submitted by FCHVs, on their community-based activities. The 230 FCHVs oriented on PPFP managed to reach 83.3% (1559) of the total number of registered pregnant mothers. They counseled them about different methods of PPFP along with other general information on safe pregnancy and childbirth in a period of two months. Among the mothers counseled, 5% chose to receive a PPIUD when they gave birth in facilities in Province One.

**Table 4:** Proportion of mothers at different stages of pregnancy counseled by FCHVs in the communities and uptake of PPIUD in facilities

Description	Number	Percent
Total FCHVs	230	
Total pregnant mothers in the catchment areas of FCHVs	1872	
Mothers counseled by FCHV on PPFP	1559	83.3
Uptake of PPIUD by the mothers counseled by FCHV	80	5.1

#### PPFP related counseling received by mothers

Table 5 demonstrates the responses from the mothers who had delivered in the two referral hospitals on the FCHV interaction and FCHV PPFP counseling they received during their pregnancy. In total 243 post-partum mothers were randomly selected for this study in the pre-intervention period and 238 post-partum mothers in the post-intervention period.

**Table 5:** Interaction and counseling coverage by FCHVs among the post-partum mothers in the two referral hospitals

Community-based counseling	Pre-intervention *n* = 243	Post-intervention *n* = 238	*P*-value
Interaction with FCHV	29 (11.9)	55 (23.2)	**0.001**
Counseling by FCHV on PPFP	17 (7)	43 (18.1)	**< 0.001**

The proportion of mothers reporting that they had interacted with FCHVs during their pregnancy in the communities was significantly higher in the post-intervention group (23.2%, *p* = 0.001) as compared to pre-intervention group (11.9%). Similarly, the proportion of mothers who have been counseled by FCHVs on PPFP methods was also significantly higher in the post-intervention group (18.1%, *p* < 0.001) as compared to the pre-intervention group (7%).

### Qualitative results

The participants of the FGD comprised of a subgroup of FCHVs who had participated in the quantitative study. In total, 54 FCHVs participated in FGD representing 6 health facilities and each FGD group had nine participants. The FCHVs represented different age groups and working experiences. The oldest FCHV was above 60 years of age and had over 25 years of working experience. The youngest FCHV was 20 years old and had working experience of less than a year.

We had five major priori themes for the six FGDs conducted. The themes helped to explain in more depth the quantitative findings on changes in their knowledge and community-based counseling activities. The first two themes focused on their knowledge of PPFP and their perceptions about the orientation program. The third and fourth themes focused on the activities they conducted after the orientation and the challenges they faced when working in the communities. The fifth theme summarized their overall impression and their suggestions for similar orientation programs and the overall role of FCHVs (Table 6).

**Table 6:** Theme from Focus Group Discussions of FCHVs

Themes	Categories	Codes
1. Knowledge on PPFP	General knowledge of PPFP	New concept Personal experience
	Applied Knowledge on PPIUD	General Knowledge Misconceptions on PPIUD Personal perspective
2. Perception of PPFP orientation program	Impression on the process	Length Content
	Usefulness	Most useful/impressive aspect
3. Activities conducted by FCHVs on PPFP	Process of Counseling coverage	Introducing PPFP
		Complimenting facility-based counseling
	Reflection of the activities	Agent of change
4. Challenges	Societal barriers	Misconceptions
		Social disparity
	Personal Fear	Personal fear
5. FCHVs’ suggestions	Process of orientation	Length of orientation
		Content delivery
		Opportunities for peer teaching/learning
	Sustainability	Involving other stakeholders
		Continuity

### Knowledge of PPFP

The majority of FCHVs suggested that PPFP was a very new concept that they only learned of through the current orientation program. Despite the general concept of FP being quite familiar for them, the majority suggested they had never heard that FP methods can be used immediately after childbirth.

“*I was very surprised that FP methods like IUD can be used immediately after birth, something I have never known or heard before in my 27 years of working as FCHV*.”-FCHV-1/Group-1.

Some FCHVs shared their personal experiences of hearing about PPFP from the mothers in the communities. They mentioned that they had never received formal orientation on PPFP.

*“I had heard about women using IUD immediately after birth in some hospitals, but no one had actually taught me about it and didn’t know that it can be used immediately. So I used to wonder how it is possible to insert so soon after birth.”-FCHV-5/Group-3.*

On further probing, some FCHVs shared misconceptions on the insertion of PPIUD during cesarean section. The responses also reflected misinformation they had received from untrained PPFP health providers and as a result of some gaps in the orientation session.

“*I think it’s not possible for women who undergo cesarean section to use PPIUD because when a woman undergoes surgery, she is under so much pain. She cannot use the IUD through vagina because it causes so much pain. This was also something told to us by a doctor in a private hospital. We don’t remember being explained about this in the training*”-FCHV-6/Group-1.

On a personal level, many of them replied that the newly acquired knowledge on PPFP had changed their personal perspectives. While many of them had previously been apprehensive about the side effects of PPIUD, following the thorough explanation during the orientation sessions, they were now more informed and confident about this method. Some reflected that they could have helped more mothers sooner including those in their own family had they known about it earlier.

*“Had we known about PPFP and in particular about PPIUD sooner, we could have suggested it to so many women. Would have made a difference in our own lives too*.”-FCHV-5/Group-4.

### Perception of PPFP orientation program

All the FCHVs suggested that the orientation program was very effective and had helped in improving their knowledge. However, some had constructive criticisms on the process of the orientation, such as, its duration.

“*The training is very useful and effective but felt the duration of the training was a bit short. If it was longer we could have discussed in more depth in a more relaxed manner.”-*FCHV-1/Group-1.

“*The training was very useful. The duration was adequate, neither long nor short. We had enough time to discuss and learn in detail that’s why we are able to answer your questions clearly now. It was also interactive and they cleared our doubts.”-*FCHV-3/Group-2.

Some responded that the best part of the orientation was the pre- and post-tests on their knowledge about PPFP. It allowed for self-assessment, and the improved scores post-orientation had helped them internalize that they had learnt something new.

*“They also distributed the papers to assess our knowledge, we were so nervous in the beginning, but then they explained everything in detail after we filled in the questions and then they took the test again after orientation. We could answer better and when the trainer said we all passed, we were so happy and felt we accomplished something significant.”-*FCHV-4/Group-4.

### Activities conducted by FCHVs on PPFP

The FCHVs reflected upon their community-based activities following the orientation. All FCHVs reported having reached out to the pregnant mothers in the communities. On average, they counseled 7–8 pregnant mothers each, per month, on healthy pregnancy, safe childbirth and PPFP methods. The FCHVs took pride in their role as a pillar of their society and in bridging the gaps in health care.

“*We are the pillars of the community. We help spread the awareness about the PPFP as a pillar too. We counsel mothers through Ama Samuha (mothers’ group), Gaun Ghar (village-home) clinic and by meeting mothers individually through home to home visits*.”-FCHV-6/Group-1.

The FCHVs also suggested that their counseling complemented that done by the health providers in the facilities. They felt that being a point of contact for mothers in the communities helped in providing additional reassurance to those who had already received counseling in the facilities.

“*Those who go to these hospitals for antenatal care regularly are counseled about different methods of PPFP including PPIUD, when they talk to us they feel more reassured. Sometimes they want a second opinion from us whether they should use it or not.”-*FCHV-3/Group-5.

All the FCHVs perceived themselves as agents of change in their communities. They believed that they were able to influence the PPFP perspectives of the people in the communities. Their responses also reflected the social commitment and sense of responsibility ingrained in them.

“*As the name suggests, we are the Swayam Sewikas (volunteers). That’s what the government suggests too. Whenever we learn something new, we just can’t wait to spread the message in the communities. Sometimes when I see a pregnant woman pass by my house, I stop cooking and run towards her and share new information.”*FCHV-3/Group-4.

### Challenges

Despite all the positive feedback they had about PPFP and the positive work they have been doing in the communities, many of them also reflected on the challenges they face. Some of the challenges were directly related to PPFP whereas others were more general.

The biggest barrier the FCHVs perceived were the deep-rooted misconceptions and beliefs that people in the communities have in general about modern contraceptives. Though they try to change people’s perspectives, they believe that they are not always able to convince the women.

*“The major challenge we often face is that many women have this misconception that temporary methods can cause cancer.”-*FCHV-4/Group-4.

The FCHVs also shared that although their voluntary role is well acknowledged by the government and most people; disadvantaged people tend to rely on them more for information, whereas people with higher social status avoid listening to them. The FCHVs believed that this puts disadvantages to those women as they have less knowledge and reduced access to free services.

*“It is easier for us to reach out to more disadvantaged and poorer communities. They are more receptive. The toughest communities to reach are the people who think they are superior, are better off and have some level of education. The sad part is that they are too prejudiced to approach us to understand about free services but also cannot afford to pay for contraceptives in private medical stores. So they are at a disadvantage*.”-FCHV-2/Group4.

Some FCHVs reflected their personal fears in approaching mothers for counseling. They shared that there have been past instances of community members forcing them to take responsibility for complications they had experienced in the health facilities. They feared similar episodes might re-occur if the mothers they had counseled faced complications with the PPIUD.

*“I am a bit worried that after we counsel these women to use PPIUD, if they decide to use it and face some complication they would come and scold us. There has not been such an incident yet. But you know as FCHVs sometimes there are ignorant people who tend to threaten us for every health consequence they face. Sometimes they say you tend to preach a lot, are you going to take the responsibility!! So that’s a concern that I have with the use of PPIUD.”-*FCHV-5/Group-3.

### FCHVs’ suggestions

The FCHVs also provided suggestions for the implementers and other stakeholders on improving the FCHV orientation and community linkages.

Most suggested an increase in the real-time demonstrations of the entire process of PPIUD insertion, they specifically suggested showing an audiovisual aid or using a dummy to show insertion techniques.

*“Showing us a video during the orientation about the details of PPFP methods and insertion of PPIUD would have been more effective.”-FCHV-3/Group2.*

While some had active ideas on the list of things to do to enhance the activities, a few of them, in particular, the older FCHVs with over 20 years of experience shared their words of wisdom. They believed that it takes time to bring change in the community and suggested that if activities are continued un-interruptedly, it may be more effective.

“*It takes time. It’s not a rapid process. When the awareness spreads gradually, the impact would be higher.”-*FCHV1/Group1.

“*Anything new will take time for people to understand. So it will take time. The concept of PPFP especially PPIUD is still new so we must be patient.”-*FCHV5/Group4.

Many of the FCHVs recommended involving wider stakeholders beyond health workers. They suggested involving local leaders, representatives of mothers’ groups and wider family members in dissemination and orientation activities. They believed such involvement would allow for a wider reach, enable task-shifting and increase awareness.

*“To make this program more effective, the program implementers should also be present in the Gaun Ghar (village-home) clinics to provide the orientation of PPFP. Direct presence would help reach out to more women. Or if not you, maybe some representatives from the health centers could also help. If some authorized people could tell these women that FCHVs are telling the truth and their counseling is effective, then the women in the communities could be more convinced.”-*FCHV-3/Group-5.

## Discussion

In this study, the intervention improved FCHV’s knowledge of PPFP significantly. After the intervention, the FCHVs were able to provide wide coverage of counseling on PPFP to women at different stages of pregnancy in the communities. Within two months of the intervention, the proportion of post-partum mothers reporting that they were counseled by FCHVs during their pregnancy improved significantly. The qualitative findings provided more depth into FCHVs’ knowledge, their impression of the intervention and further details about the activities and challenges they faced.

The quantitative findings suggested that the knowledge of PPFP among FCHVs improved significantly after the intervention. Qualitative findings supported this and the majority of mothers were able to explain PPIUD. A previous qualitative study from the same initiative had identified the lack of knowledge among FCHVs as an important barrier to women’s uptake of immediate PPFP methods [11]. FCHVs are often the first point of contact for women in the communities to seek correct information and timely health care. Thus, the findings from this study are encouraging as the improved knowledge of PPFP among FCHVs would enable them to provide correct and timely information to the women in need of PPFP services.

The qualitative findings indicated that the majority of FCHVs perceived the intervention to be useful. However, some FCHVs also highlighted certain gaps in the intervention process. Some FCHVs wished that the duration of the intervention was longer and more interactive, while some reflected on the lack of use of audiovisual aids such as videos on PPFP methods and in particular on PPIUD insertion. It was a conscious decision made by the implementers to simplify the content delivery considering the limited education levels among FCHVs. It indicates the need for the implementers not to under-estimate use of technology when interacting with people with limited literacy. Studies suggest that interactive sessions with adequate time provide more knowledge retention [25]. Thus, the feedback from the FCHVs is important to take into consideration to improve the delivery of the intervention in the future.

In this study, both the quantitative and qualitative findings suggested that FCHVs were able to reach out to many mothers at different stages of pregnancy in the communities and counsel them on PFPP. Though FCHVs have managed to conduct many MNCH related activities in the past [19–21], PPFP related activities were almost non-existent before the intervention. With the improved knowledge of PPFP, FCHVs were able to encourage women to go to facilities for childbirth [21] and also inform them about the contraceptive choices available immediately after giving birth in facilities.

FCHVs reported that 5% of the pregnant women they had counseled had chosen PPIUD when they gave birth in various hospitals including the two study hospitals in Morang District, Province One. Nepal does not have specific PPIUD nationally representative data, however, the overall uptake of IUD was found to be just 1.4% [13]. Therefore, the early findings of the effect of FCHV’s involvement in PPFP is quite encouraging.

The quantitative findings also found an increased proportion of mothers delivering in the hospitals had been counseled by FCHVs during their pregnancy. This finding validates the community-based activities conducted by the FCHVs by reflecting their work in the experiences of women delivering at the facilities. Though still early to assess the longer term impact of the intervention, this finding indicates that a significant proportion of mothers reaching the health facilities for childbirth are already informed about the PPFP options they can choose after the childbirth. The increase in institutional delivery provides an increased opportunity for women to receive PPFP services such as PPIUD [12]. This enables women to receive both obstetric care and PPIUD services in the same setting around the same time. Thus, FCHVs role in PPFP provides an opportunity to increase the demand and access to PPFP services in the health facilities.

The qualitative findings also provided more information about the challenges FCHVs face while working in the communities. Some challenges were similar to those faced by community health workers (CHW) in other countries, such as Brazil, which included lack of understanding among the people and lack of respect of their work in communities [26]. FCHVs in this study, also pointed out the social barriers and deep-rooted misconceptions among the mothers about modern contraceptives making it difficult for them to convince the people in the communities of their benefits. Studies from LMICs have indicated multiple barriers among mothers on the uptake of PPFP methods, such as PPIUD, which include social taboos, lack of knowledge, lack of autonomy to make decisions and many more [4–6]. As indicated by some FCHVs in the FGD, change could take time and consistency in providing community awareness could be the most important solution to overcome barriers related to PPFP in communities.

Overall, this study indicates that the intervention has helped in improving FCHVs’ knowledge on PPFP and enabled them to include PPFP counseling in their MNCH related activities in the communities. A systematic review on CHW’s role in FP services in LMIC has indicated positive results in improving the use of modern contraception and in improving knowledge and attitude related to FP [27]. However, these studies have focused on FP in general and have not explored the roles of CHW specifically in PPFP. This study provides important baseline information on CHW’s role in PPFP.

### Limitations

This study has certain limitations. Firstly, the length of the study is too short due to the time limitation of the initiative. A longer duration would have provided an opportunity to carefully follow-up the FCHVs to evaluate any changes in their knowledge and behaviors related to community-based counseling in the longer run. Moreover, it could have provided enough time to assess the long-term impact of the FCHV’s role in changing PPFP related intention and behaviors of mothers. Secondly, this study lacked control groups of FCHVs who had not received the orientation program. Having a control group for pre- and post-intervention periods could have provided a better comparative perspective. Thirdly, this study does not have information from other stakeholders involved in the intervention, such as, the health providers who were involved as facilitators in the orientations. Furthermore, no data related to the coverage of the facility-based counseling in the peripheral health posts was available. This information could have provided a more complete picture of the roles at each layer of the health system.

Despite the limitations, this is the first study that has assessed the role of FCHV in delivering community-based PPFP counseling services in Nepal. The findings indicate that involving FCHVs in PPFP programs is beneficial and they also indicate the inadequacies in existing PPFP programs. Although PPFP was introduced in Nepal over a decade ago, it is still a new concept for FCHVs; this reflected the lack of community-level interventions and awareness activities. Despite the short period of time, these FCHVs were able to reach out to a large number of mothers. As for many other health interventions, FCHVs could be a sustainable medium for community mobilization for PPFP programs. Follow-up studies are necessary to assess the long term impact of such interventions in the communities and similar studies involving CHW from other LMICs could provide a global perspective.

## Conclusion

This study showed that the orientation of PPFP improved the knowledge of FCHVs on PPFP which in turn improved their counseling coverage on PPFP in the communities. The orientation improved the FCHV’s knowledge of PPFP and the quality of their community-based counseling. The increased proportion of mothers being counseled by FCHVs has also been reflected in the facility-based results. Longer follow-up studies are warranted to provide a longitudinal perspective on the effect of FCHV’s community-based activities on mother’s uptake of PPIUD.

## Supplementary information

**Additional file 1.** Interview Questionnaire for Female Community Health Volunteers.

**Additional file 2.** Checklist for FCHVs monthly reporting forms.

**Additional file 3.** Interview Questionnaire for post-partum mothers.

**Additional file 4.** FGD checklists for FCHVs.

## Data Availability

All anonymized quantitative data will be available in Figshare repository upon acceptance of the manuscript. Qualitative data will be available from the corresponding author only upon reasonable request as it contains personal quotes from the respondents which maybe identifiable.

## Abbreviations

CHW: Community health workers
DCO: Data collection officer
FCHV: Female Community Health Volunteer
FGD: Focus group discussions
FIGO: International Federation of Gynecology and Obstetrics
FP: Family planning
IUD: Intrauterine contraceptive device
KZH: Koshi Zonal Hospital
LMIC: Low and middle income countries
MNCH: Maternal newborn and child health
MOHP: Ministry of Health and Population
NESOG: Nepal Society of Obstetricians and Gynecologists
NHTC: National Health Training Center
NMCTH: Nobel Medical College Teaching Hospital
PPFP: Post-partum family planning
PPIUD: Post-partum intrauterine contraceptive device

## References

[1] Rossier C, Bradley SE, Ross J, Winfrey W (2015). Reassessing unmet need for family planning in the post-partum period. Stud Fam Plan.

[2] Mehata S, Paudel YR, Mehta R, Dariang M, Poudel P, Barnett S (2014). Unmet need for family planning in Nepal during the first two years post-partum. Biomed Res Int.

[3] World Health organization (2013). Programming strategies for Post-partum Family Planning.

[4] Panday S, Bissell P, van Teijlingen E, Simkhada P (2017). The contribution of female community health volunteers (FCHVs) to maternity care in Nepal: a qualitative study. BMC Health Serv Res.

[5] Abdel-All M, Putica B, Praveen D, Abimbola S, Joshi R (2017). Effectiveness of community health worker training programmes for cardiovascular disease management in low-income and middle-income countries: a systematic review. BMJ Open.

[6] Ndayizigiye M, Fawzi MC, Lively CT, Ware NC (2017). Understanding low uptake of contraceptives in resource-limited settings: a mixed-methods study in rural Burundi. BMC Health Serv Res.

[7] Kapp N, Curtis KM (2009). Intrauterine device insertion during the post-partum period: a systematic review. Contraception..

[8] Goldstuck ND, Steyn PS (2017). Insertion of intrauterine devices after cesarean section: a systematic review update. Int J Women's Health.

[9] National Collaborating Centre for Women’s and Children’s Health (UK) (2005). Long-acting Reversible Contraception: The Effective and Appropriate Use of Long-Acting Reversible Contraception.

[10] Makins A, Arulkumaran S (2018). Institutionalization of post-partum intrauterine devices. Int J Gynaecol Obstet.

[11] Thapa K, Dhital R, Rajbhandari S, Acharya S, Mishra S, Pokhrel SM (2019). Factors affecting the behavior outcomes on post-partum intrauterine contraceptive device uptake and continuation in Nepal: a qualitative study. BMC Pregnancy Childbirth.

[12] Makins A, Taghinejadi N, Sethi M, Machiyama K, Thapa K, Perera G (2018). Factors influencing the likelihood of acceptance of post-partum intrauterine devices across four countries: India, Nepal, Sri Lanka, and Tanzania. Int J Gynaecol Obstet.

[13] Ministry of Health, New Era, ICF (2017). Nepal Demographic and Health Survey 2016.

[14] Thapa K, Dhital R, Karki YB, Rajbhandari S, Amatya S, Pande S (2018). Institutionalizing post-partum family planning and post-partum intrauterine device services in Nepal: role of training and mentorship. Int J Gynaecol Obstet.

[15] Khatri RB, Mishra SR, Khanal V (2017). Female community health volunteers in community-based health programs of Nepal: future perspective. Front Public Health.

[16] Neupane D, McLachlan CS, Mishra SR, Olsen MH, Perry HB, Karki A (2018). Effectiveness of a lifestyle intervention led by female community health volunteers versus usual care in blood pressure reduction (COBIN): an open-label, cluster-randomised trial. Lancet Glob Health.

[17] McPherson RA, Tamang J, Hodgins S, Pathak LR, Silwal RC, Baqui AH (2010). Process evaluation of a community-based intervention promoting multiple maternal and neonatal care practices in rural Nepal. BMC Pregnancy Childbirth..

[18] Rajbhandari S, Hodgins S, Sanghvi H, McPherson R, Pradhan YV, Baqui AH (2010). Expanding uterotonic protection following childbirth through community-based distribution of misoprostol: operations research study in Nepal. Int J Gynaecol Obstet.

[19] Neupane D, Dawson P, Houston R, Dhakal L, Sharma J, Gargi KC (2017). Lower mortality is observed among low birth weight young infants who have received home-based care by female community health volunteers in rural Nepal. BMC Pregnancy Childbirth..

[20] Amano S, Shrestha BP, Chaube SS, Higuchi M, Manandhar DS, Osrin D (2014). Effectiveness of female community health volunteers in the detection and management of low-birth-weight in Nepal. Rural Remote Health.

[21] Dhital R, Silwal RC, Simkhada P, Teijlingen EV, Jimba M (2019). Assessing knowledge and behavioural changes on maternal and newborn health among mothers following post-earthquake health promotion in Nepal. PLoS One.

[22] Creswell JW, CP (2010). Choosing a mixed method design 2nd edition ed.

[23] Dhand, N. K., & Khatkar, M. S. (2014). Statulator: An online statistical calculator. Sample Size Calculator for Comparing Two Independent Proportions. Accessed 18 August 2019 at http://statulator.com/SampleSize/ss2P.html

[24] Vaismoradi M, Turunen H, Bondas T (2013). Content analysis and thematic analysis: implications for conducting a qualitative descriptivestudy. Nurs Health Sci.

[25] Robinson N, Moshabela M, Owusu-Ansah L, Kapungu C, Geller S (2016). Barriers to intrauterine device uptake in a rural setting in Ghana. Health Care Women Int.

[26] Grossman-Kahn R, Schoen J, Mallett JW, Brentani A, Kadelitz E, Heisler M (2018). Challenges facing community health workers in Brazil's family health strategy: a qualitative study. Int J Health Plann Manag.

[27] Scott VK, Gottschalk LB, Wright KQ, Twose C, Bohren MA, Schmitt ME (2015). Community health Workers' provision of family planning Services in low- and Middle-Income Countries: a systematic review of effectiveness. Stud Fam Plan.

