# Supplementary material for: Improving post-partum family planning services provided by female community health volunteers in Nepal: a mixed methods study
Source: BMC Health Serv Res. 2020 Feb 17;20:123. doi: 10.1186/s12913-020-4969-1 (PMC7027278; doi:10.1186/s12913-020-4969-1)
Supplement: Supplementary file 1 — Additional file 1. Interview Questionnaire for Female Community Health Volunteers. [file 12913_2020_4969_MOESM1_ESM.docx]

**Additional file 1: Interview Questionnaire for Female Community Health Volunteers**

**(To be filled by the health providers in peripheral facilities)**

**Interview date:…………………………… Interview by:………………………………**

**Name of the health facility………………….**

**Name of the FCHV…………………..**

**Orientation status 1. Pre- orientation 2. Post-orientation**

**General information**

|  | **Questions** | **Responses** |
| --- | --- | --- |
|  | How old are you? | …………………..yrs |
|  | What is your highest level of education? | 1. Can read and write 2. Primary level (grade 1 to 8) 3. Secondary level (grade 8 to 12) 4. University level (bachelors degree and above) |
|  | How long have you been working as FCHV? | …………..yrs |
|  | In which area do you currently work as FCHV? | Province….  District……..  Palika……  Ward………. |
|  | Have you ever attended orientation on family planning? | 1. Yes 2. No |
|  | Have you ever attended orientation on post-partum family planning in the past? | 1. Yes 2. No |
|  | Have you ever attended orientation on PPIUCD in the past? | 1. Yes 2. No |

**Knowledge on PPFP/ PPIUCD**

|  | **Questions** | **Responses** |
| --- | --- | --- |
|  | Immediately after delivery, women cannot use contraception | 1. True 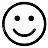 2. False 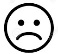 |
|  | Postpartum Intrauterine devices can provide protection up to twelve years | 1. True 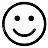 2. False 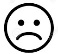 |
|  | Women who undergo a caesarean section can have postpartum IUCD inserted | 1. True 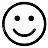 2. False 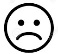 |
|  | IUCDs can be inserted immediately after giving birth | 1. True 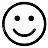 2. False 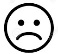 |
|  | If IUCD strings are seen outside vagina, they should go for follow up immediately | 1. True 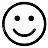 2. False 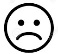 |
