# Supplementary material for: Improving post-partum family planning services provided by female community health volunteers in Nepal: a mixed methods study
Source: BMC Health Serv Res. 2020 Feb 17;20:123. doi: 10.1186/s12913-020-4969-1 (PMC7027278; doi:10.1186/s12913-020-4969-1)
Supplement: Supplementary file 2 — Additional file 2. Checklist for FCHVs monthly reporting forms. [file 12913_2020_4969_MOESM2_ESM.docx]

**Additional file 4: Checklist for FCHVs monthly reporting forms**

**Date of checklist collected:…………..**

**Collected by:……………**

**FCHV ID NO:.………..**

**Name of the health facility:…………….**

|  | **Month-1** | **Month-2** |
| --- | --- | --- |
|  |  |  |
| **Number of pregnant women in the community** |  |  |
| **Number of women delivered in the community** |  |  |
| **Number of women counseled on PPFP** |  |  |
| **Number of women using PPIUD** |  |  |
