# Supplementary material for: Improving post-partum family planning services provided by female community health volunteers in Nepal: a mixed methods study
Source: BMC Health Serv Res. 2020 Feb 17;20:123. doi: 10.1186/s12913-020-4969-1 (PMC7027278; doi:10.1186/s12913-020-4969-1)
Supplement: Supplementary file 3 — Additional file 3. Interview Questionnaire for post-partum mothers. [file 12913_2020_4969_MOESM3_ESM.docx]

**Additional file 2:** **Interview Questionnaire for postpartum mothers**

**Registration**

**INTRODUCTION by the DCOs to the participant**

- - 1. Respondent ID Generation – (ID number generated in the app)
    2. Facility Name
       1. Koshi Zonal Hospital, Biratnagar
       2. Nobel Medical College Teaching Hospital
    3. DCO number * *(an identification number is provided for each DCO)……………………..*
    4. Date of Interview………………………………
    5. Date of Delivery …………………………..

**FCHV related questions**

| 1. 16. | Have you ever interacted with an FCHV during your last pregnancy? | 1. Yes 2. No |
| --- | --- | --- |
| 1. 17. | If yes, where did you meet/interacted with the FCHV? | Province………..  District…………  Palika…………..  Ward…………….. |
| 1. 18. | Did the FCHV counseled you about different methods of PPFP in your last pregnancy?  Skip if never interacted with FCHV | 1. Yes 2. No |
| 1. 19. | Did the FCHV counseled you about PPIUD in your last pregnancy? Skip if never interacted with FCHV | 1. Yes 2. No |
| 1. 20. | Did the FCHV suggested you to go to the health facility to learn more about PPIUD? Skip if never interacted with FCHV | 1. Yes 2. No |
