# Supplementary material for: Improving post-partum family planning services provided by female community health volunteers in Nepal: a mixed methods study
Source: BMC Health Serv Res. 2020 Feb 17;20:123. doi: 10.1186/s12913-020-4969-1 (PMC7027278; doi:10.1186/s12913-020-4969-1)
Supplement: Supplementary file 4 — Additional file 4. FGD checklists for FCHVs. [file 12913_2020_4969_MOESM4_ESM.docx]

**Additional file 4: FGD checklists for FCHVs**

- Introduction by the research team
- Introduction of the FCHVs

**Overall perception about PPFP/PPIUD**

| - How much do you know about different methods of PPFP? |
| --- |
| - What are the methods you know? |
| - How much do you know about PPIUD? |
| - What is your perception about PPIUD? |
| - Has the perception about PPIUD changed after the orientation program? |
| -If yes, why and what are the changes in your perceptions? |
| -If No, why? |

**Perception about PPFP orientation program**

| - How did you find the orientation program? |
| --- |
| - What you were the things you liked about the orientation program? |
| - What were the things you did not like about the orientation program? |
| - What were the key messages that you learned about PPFP after the orientation program? |
| - What were the key message that you learned about PPIUD after the orientation program? |

**PPFP counseling and referral behavior**

| - Have you ever counseled any women about PPFP before you attended the orientation?   -If yes, what kind of suggestion did you give? |
| --- |
| - Have you counseled any women about PPFP after you attended the orientation?   -If yes, what kind of suggestions did you give? |
| - Have you explained about PPIUD to any women?   -If yes, what did you explain |
| - Have you referred anyone to hospital to use PPIUD?   If yes, why? And how did you refer?  If No, why? |
| - Did you come across any women with complications or problems with PPIUD?   If yes, what were the types of complications or problems you found?  And did you do to overcome the problem? |

**Recommendations**

| What are the recommendations you would like to give to the concerned stakeholders to improve such orientation programs in the future? |
| --- |

**Note: further questions will be added based on any specific findings from the quantitative study, to explain the quantitative findings better**
